# Efficacy and Prognosis of Hyperbaric Oxygen as Adjuvant Therapy for Neonatal Hypoxic-Ischemic Encephalopathy: A Meta-Analysis Study

**DOI:** 10.3389/fped.2022.707136

**Published:** 2022-04-21

**Authors:** Xiu-Bing Gong, Rui-Hua Feng, Hong-Mei Dong, Wen-Hua Liu, Ya-Nan Gu, Xiang-Yue Jiang, Ye-Hao Lou, Jun Xu, Qing-Li Dou

**Affiliations:** ^1^The Second School of Clinical Medicine, Southern Medical University, Guangzhou, China; ^2^Department of Health Economics, Institute of Medical Information, Chinese Academy of Medical Sciences and Peking Union Medical College, Beijing, China; ^3^No. 941 Hospital of the Joint Support Force of People’s Liberation Army (PLA), Xining, China; ^4^Shenzhen Bao’an People’s Hospital, Shenzhen, China; ^5^Emergency Department, The Second Affiliated Hospital of Shenzhen University, Shenzhen, China; ^6^The First School of Clinical Medicine, Guangdong Medical University, Zhanjiang, China; ^7^State Key Laboratory of Complex Severe and Rare Diseases, Emergency Department, Peking Union Medical College Hospital, Chinese Academy of Medical Sciences and Peking Union Medical College, Beijing, China

**Keywords:** neonate, hypoxic-ischemic encephalopathy, hyperbaric oxygen therapy, randomized controlled trials, meta-analysis

## Abstract

**Background:**

Preclinical and clinical evidence suggests that hyperbaric oxygen therapy (HBOT) may benefit newborns. The effectiveness of HBOT for neonatal hypoxic-ischemic encephalopathy (HIE) remains controversial. We conducted a meta-analysis to evaluate the efficacy and prognosis of HBOT in neonates with HIE.

**Methods:**

A systematic search of eight databases was performed for available articles published between January 1, 2015, and September 30, 2020, to identify randomized controlled clinical trials (RCTs) on HBOT for neonatal HIE. Methodological quality assessment was performed by applying the simple procedure detailed by the Cochrane collaboration. Afterward, quality assessment and data analysis were performed using Revman 5.3 software. STATA 15 software was used to detect publication bias as well as for sensitivity analysis.

**Results:**

A total of 46 clinical RCTs were selected for the study and included 4,199 patients with neonatal HIE. The results indicated that HBOT significantly improved the total efficiency (TEF) of treatment for neonatal HIE patients [odds ratio (OR) = 4.61, 95% confidence interval (CI) (3.70, 5.75), *P* < 0.00001] and reduced the risk of sequelae (OR = 0.23, 95% CI (0.16, 0.33), *P* < 0.00001) and the neonatal behavioral neurological assessment (NBNA) scores [mean difference (MD) = 4.51, 95%CI (3.83,5.19, *P* < 0.00001)].

**Conclusion:**

In light of the effectiveness of HBOT neonatal HIE, this meta-analysis suggested that HBOT can be a potential therapy for the treatment of neonatal HIE. Due to the heterogeneity of studies protocol and patient selection being only from China, more research is needed before this therapy can be widely implemented in the clinic.

**Protocol Registration:**

PROSPERO (ID: CRD42020210639). Available online at: https://www.crd.york.ac.uk/prospero/display_record.php?ID=CRD42020210639.

## Introduction

Neonatal hypoxic-ischemic encephalopathy (HIE) is a clinical syndrome caused by long-term cerebral hypoxia and ischemia in premature or full-term infants before or after birth due to placenta loss, umbilical cord prolapse, and uterine rupture (1). Neonatal acute brain injury (2) is characterized by long-term neurological dysfunction due to perinatal hypoxic ischemia, leading to a high incidence of sequelae and mortality (3). Studies have shown that neonates who died of hypoxic-ischemic encephalopathy (HIE) accounted for up to 23% of neonatal deaths (4–6). In developed countries, the incidence is approximately 1.5 cases per 1,000 live births; however, a higher incidence of 10–20 cases per 1,000 live births is observed in lower- and middle-income countries (7).

Neonates with HIE experience seizure activity, cranial nerve dysfunction (e.g., weak or absent suck reflex), impaired motor ability, and altered mental state. Neonatal HIE is categorized as mild, moderate, or severe based on the symptoms using a modified scoring system (8, 9). Hence, it is essential first to understand the HIE stage and use the proper treatment to ensure a successful treatment outcome. Neuroprotective drugs such as melatonin, allopurinol, topiramate, erythropoietin, and mild hypothermia are effective treatment measures (10). At present, the gold standard treatment for neonates with moderate to severe HIE includes the application of moderate hypothermia (HT; a decrease in temperature by 2–5^°^C), where the body temperature is maintained at 33.5^°^C for up to 72 h within the first 6 h of life (11). The goal of hypothermia is to slow cerebral metabolism, reduce reperfusion injury, and prevent neuronal apoptosis. Hypothermia therapy does not increase damage to other tissues and organs and has matured to treat cerebral hemorrhage, cerebral ischemia, and cerebral hypoxia (12). However, inducing hypothermia for a longer period and applying deeper cooling has not shown any additional benefits.

Similarly, for patients with severe HIE, minimal hypothermia is not appropriate to treat neurodevelopment disabilities. In a recent review, the authors concluded that hypothermia is effective only for a small number of neonates; however, it is still considered one of the most significant recent innovations for the treatment of HIE (13). Hence, we can conclude that the effect of conventional treatment is limited mainly because HIE is not a single disease entity but a condition with diverse causes that manifest as signs of brain injury, and this disease possesses multifactorial etiopathogenesis. Consequently, there are numerous contraindications, multiple side effects, and unclear pharmacological mechanisms for the available drugs (10).

In recent years, researchers have suggested that appropriate treatments can improve newborn HIE. Hyperbaric oxygen therapy (HBOT) has a particularly good effect on hypoxic diseases, can improve the survival rate of neonatal HIE, and has the potential to improve neurological dysfunction, reducing the incidence of sequelae (14). HBOT is achieved when a patient inhales 100% oxygen inside a hyperbaric chamber pressurized to a value greater than 1 atmosphere (atm). HBOT can reduce the tissue damage from brain edema caused by ischemia and hypoxia by improving tissue oxygenation, increasing the cerebral blood flow, promoting vascular repair, enhancing angiogenesis, and decreasing inflammation, thereby promoting neurological recovery through the regeneration of the axial white matter and improving patient prognosis and survival (15, 16). These chronological events can gradually improve stunted areas of the brain and the metabolic function through the activation of neuroplasticity (16). The efficiency of HBOT was examined in a recent randomized control trial (RCT) on stroke patients. The researchers observed enhanced perfusion in the regions with low living cell activity, among which the diencephalon was a major area. As cortico-thalamic projections regulate the network function, the researchers hypothesized that improved diencephalon perfusion with HBOT may contribute to the recovery of consciousness (17). Zhou et al. (18) confirmed that HBOT could reduce the mortality and disability rate of neonatal HIE. In this study, neonates with HIE were treated with HBOT at 1.4 ATA, 1.5 ATA, and 1.6 ATA of pressure. Serum superoxide dismutase (SOD), nitric oxide synthase (NOS), malondialdehyde (MDA), and nitric oxide (NO) levels were measured on Days 1 and 7 after HBOT. The authors observed an elevation in the serum SOD and a reduction in NOS, MDA, and NO levels after HBOT, confirming that as the hyperbaric oxygen pressure increased, the antioxidant capacity was enhanced. HBOT has been identified as an effective treatment for ischemic injuries in the central nervous system. Several animal studies have also confirmed that HBOT can alleviate hypoxic-ischemic brain injury and significantly improve neurological function deficits in neonatal rats (19–21). In an *in vivo* rat model of permanent middle cerebral artery occlusion (MCAO), it was found that HBOT lowered the infarct volume and improved the neurological scores in the injured rats (22). Although some studies have reported that HBOT is effective for neonatal HIE, the findings lack further confirmation with large sample sizes, multi-center studies, and randomized clinical studies. Therefore, this meta-analysis aimed to comprehensively analyze previous clinical RCTs involving HBOT for neonatal HIE to facilitate more reliable, clinical, evidence-based medicine.

## Materials and Methods

This systematic review was conducted and reported according to the instructions provided in the Preferred Reporting Items for Systematic Reviews and Meta-Analyses (PRISMA) guidelines (23).

### Search Strategy

Several articles were found during an initial literature search with no restrictions on the year, but only studies published from 2003 to 2020 were considered. We extensively searched various types of published literature using several databases. RCTs on HBOT for neonatal HIE were retrieved from eight databases: PubMed, EMBASE, Cochrane Library, Web of Science, Chinese Biomedical (CBM), Chinese Science and Technology Periodical Database (VIP), WanFang, and China National Knowledge Infrastructure (CNKI). The electronic search strategy for HBOT on neonatal HIE was “neonate” or “neonatal infant” and “hyperbaric oxygenation therapy” or “oxygen therapies, hyperbaric” and “hypoxic-ischemic encephalopathy” and “RCTs.” There was no language limit. The references for the retrieved articles were examined to identify relevant studies. The Cochrane Collaboration database was also searched, and a “cited reference search” was performed to identify articles citing the retrieved studies. The author data were reviewed for relevant articles.

### Eligibility Criteria

We included RCTs that reported HBOT for neonatal HIE. It was mandatory to follow each step of the protocol meticulously. If there were any deviations from the study protocol, the committee would not approve the study. The reviewers insisted on checking the primary safety and efficacy endpoints of the treatment strategy.

### Inclusion Criteria

(i) Clinical RCTs conducted with and without a blinding strategy were included in the study literature search.
(ii) Children who met the HIE diagnostic criteria confirmed with obstetric history, neurological symptoms of the newborn, computed tomography, ultrasound, or magnetic resonance imaging (MRI) were included in the study. Children were excluded from the study if they had any other encephalopathies.
(iii) The experimental group was treated with HBOT in addition to conventional treatment. The conventional treatment (CT) group was treated with medication, oxygen inhalation, spasm relief measures, and the correction of water and electrolyte imbalance.

### Exclusion Criteria

(i) Non-RCTs were not considered for the study
(ii) Absence of a control group in the study
(iii) Experiments on animals (*in vivo*)
(iv) Repeated publication of data, a compilation of letters, case reports, reviews, and systematic evaluations
(v) Literature consisting of incomplete data

### Data Extraction

Research studies, review articles, other systematic review articles, and articles without information on HBOT for neonates with HIE were excluded. Study reports were thoroughly screened again and rechecked by two independent reviewers to determine whether the articles matched the inclusion and exclusion criteria. Two reviewers were paired up based on their educational background to ensure the pair contained at least one person with clinical expertise and one person with research experience. The two reviewers independently reviewed the same research articles that met the criteria and retrieved the articles for a full review independently. The following data were extracted: (i) General trial characteristics (first author’s surname, publication date, and study time); (ii) Baseline characteristics of the patient and clinical data (number and age of patients per group); (iii) Intervention measures (duration and dose of HBOT). If any discrepancies regarding the eligibility criteria of the studies were found, they were brought to the notice of the full study team for a final decision. Additional articles were also reviewed based on the reference lists, and appropriate information was obtained. Again, the reviewers cross-checked all the articles, obtained a final list of references, and discussed the data with a third reviewer for the meta-analysis.

### Quality Analysis

The quality of the included articles was evaluated by two of the authors independently. Disagreements were resolved through discussion or consultation with a third author. The quality of the included studies was assessed based on the Cochrane Reviewers’ Handbook 5.1.0 RCT risk assessment tool (24). The following seven items were evaluated: (i) The random sequence generation method; (ii) The allocation hiding mechanism; (iii) Whether the operators/patients were blinded; (iv) Whether the evaluator of the results was blinded; (v) Whether the data were complete; (vi) Whether there was selective reporting; (vii) Other bias risks.

### Outcome Indicators

Outcome indicators for our study are the following:

(i) Total efficiency (TEF) (25): The efficacy was evaluated according to the children’s clinical symptoms, signs, and craniocerebral computed tomography manifestations. Significantly effective: the child becomes awake after treatment, the pre-halogen tension, muscle tension, and breathing conditions return to normal, the pupils are of equal size, and there is no symptom of twitching, and the child can have grasping and hugging reflexes after guidance. The brain CT results showed no abnormalities. Invalid: After the treatment, the symptoms and signs of the child are still abnormal, or the brain CT results suggest an abnormal condition.
(ii) Risk of sequelae: Sequelae can reflect the prognosis of the treatment, including hydrocephalus, epilepsy, cerebral palsy, linguistic intelligence, and physical development.
(iii) Neonatal behavioral, neurological assessment (NBNA) (26): The NBNA score has a total of 20 items in 5 parts, including four active muscle tension, four passive muscle tension, four original reflexes, four behavioral abilities, and four general evaluations. Each item is worth 4 points, 40 points in total, < 35 Score means abnormal, ≥ 35 means normal. The higher the score, the better the behavioral, neurological ability of the child.

### Statistical Analysis

The Reviewer Manager 5.3.5 software was used to conduct statistical analysis on the extracted data. Dichotomous data were expressed as odds ratio (OR) and 95% confidence interval (CI), whereas the continuous data were presented as mean difference (MD) with 95%CI. The data heterogeneity was associated with a combination of the fixed-effects (FE) and random-effects (RE) models, and the chi-square test was used to determine the heterogeneity. A fixed-effects and a random-effects model were used to merge the data according to heterogeneity, which was determined using the chi-square test. The *I*^2^ statistic, an *I*^2^ < 25%, indicates that heterogeneity may not be important, a value between 25 and 50% represents moderate inconsistency, and *I*^2^ > 50% suggests severe heterogeneity. We defined *P* ≥ 0.1 and *I*^2^ < 50 as an indicator that the results have a good agreement and that the fixed-effects model (FEM) may be set, while *I*^2^ > 50% was defined as an indicator of striking heterogeneity between the data (27). Otherwise, the RE model was employed to pool the results to minimize potential clinical heterogeneity. STATA 15.1 was used for sensitivity analysis to detect the possible sources of significant heterogeneity. Publication bias was evaluated using Egger’s test. A *P*-value < 0.05 suggested that there was publication bias.

## Results

### Literature Inclusion and Quality Evaluation

**FIGURE 1:**
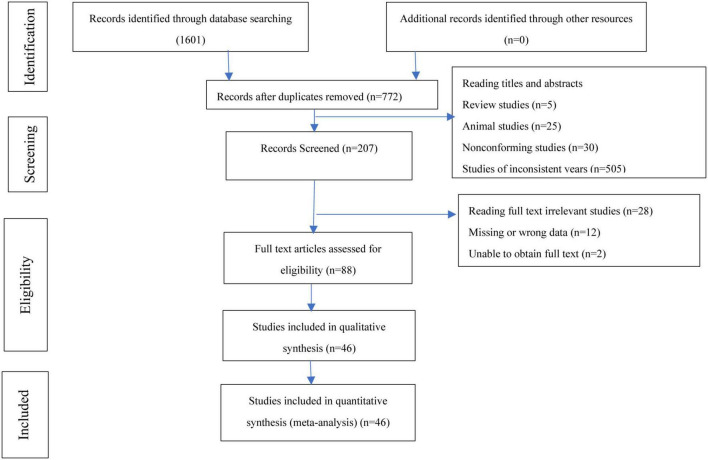
Flow diagram of study selection.

We found similar studies close to our research topic during the search, but we focused only on our selected MESH terms and retrieved the full-text articles relevant to our study. We also identified additional studies from the reference lists during the literature search, and 1,601 eligible studies were finally retrieved from the eight databases used. A total of 46 clinical RCTs that matched the inclusion/exclusion criteria were included (28–73). All of the trials were performed in China. The characteristics of each article’s literature were identified and are shown in Figure 1. Additional information is presented in Table 1, with detailed information on each of the studies included.

**TABLE 1:** Basic features of the studies included in the meta-analysis.

Study	Study period	Sample size T (E/C)	Age (d)/Gestational age (w) E/C	Intervention E*	C	Treatment duration
Li et al. (52)	2013–2017	130 (65/65)	13.54 ± 3.83 (d)/13.22 ± 3.11 (d)	CT + HBOT (60 min, qd, 1.6 ATA)	CT	90 days
Tian et al. (59)	2016–2019	61 (30/31)	39.11 ± 2.02 (w)/39.11 ± 2.02 (w)	CT + HBOT (60 min, qd, 0.04–0.05 MPa)	CT	10 days
Yan et al. (63)	2013–2014	98 (49/49)	3.6 ± 0.1 (d)/3.6 ± 0.1 (d)	CT + HBOT (60 min, qd, 1.3∼1.6 ATA)	CT	10 days
Jiang (50)	2015–2016	62 (31/31)	4.1 ± 1.4 (d)/4.3 ± 1.2 (d)	CT + HBOT (35 min, qd, –)	CT	30 days
Jia and Guo (49)	2013–2014	92 (46/46)	3.2 ± 0.2 (d)/3.1 ± 0.1 (d)	CT + HBOT (35 min, qd)	CT	30 days
Weng (62)	2014–2017	92 (46/46)	2.3 ± 0.1 (d)/2.1 ± 0.2 (d)	CT + HBOT (60 min, qd, 0.04∼0.06 MPa)	CT	–
Zhang et al. (73)	2014–2015	87 (49/38)	32 ± 3 (w)/33 ± 3 (w)	CT + HBOT (70 min, qd, 0.04–0.06 MPa)		10 days
Chen (67)	2010–2016	40 (20/20)	–/–	CT + HBOT (60 min, qd, 0.04 MPa)	CT	–
Wang (39)	2016	74 (37/37)	37 ± 4.2 (w)/37 ± 4.2 (w)	CT + HBOT (60 min, qd, 0.06–0.08 MPa)	CT	14–28 days
Jiang et al. (35)	–	240 (120/120)	37.0 ± 11.5 (w)/36.4 ± 10.6 (w)	CT + HBOT (60 min, qd, –)	CT	21 days
Zhou (68)	2013–2014	120 (60/60)	37.2 + 1.4 (w)/36.5 + 1.4 (w)	CT + HBOT (60–90 min, qd, 0.04 MPa)	CT	56 days
Deng and Liu (44)	2013–2015	86 (43/43)	3.1 + 0.3 (d)/3.2 + 0.2 (d)	CT + HBOT (35 min, qd, –)	CT	30 days
Jin (36)	2012–2013	60 (30/30)	38.5 ± 0.7 (w)/38.5 ± 0.7 (w)	CT + HBOT (50 min, qd, 0.04∼0.06 MPa)	CT	30–90 days
A (90)	2017–2019	60 (30/30)	3.59 ± 0.25 (d)/3.52 ± 0.23 (d)	CT + HBOT (90 min, qd, 0.05∼0.07 MPa)	CT	30 days
Han (33)	2010–2013	100 (50/50)	38.97 ± 1.12 (w)/38.94 ± 1.10 (w)	CT + HBOT (60 min, qd, 0.05∼0.07 MPa)	CT	30–60 days
Yang (64)	2015–2016	80 (40/40)	11.21 + 0.1 (d)/14.21 ± 0.1 (d)	CT + HBOT (60 min, qd, 0.05–0.06 MPa)	CT	–
Fang (46)	2013–2014	72 (36/36)	38.6 ± 1.3 (w)/38.1 ± 1.2 (w)	CT + HBOT (60 min, qd, 0.04 MPa)	CT	7–28 days
Liu and He (38)	2013–2015	80 (40/40)	37.1 ± 0.8 (w)/37.4 ± 0.6 (w)	CT + HBOT (50 min, qd, 0.03–0.04 MPa)	CT	30 days
Shu (58)	2016–2018	96 (48/48)	40.0 ± 2.1 (w)/39.5 ± 2.2 (w)	CT + HBOT (60 min, qd, < 0.1 MPa)	CT	60 days
Liu et al. (53)	2013–2014	59 (30/29)	39 ± 2.33 (w)/39 ± 3.12 (w)	CT + HBOT (–, qd, < 0.1 MPa)	CT	20–30 days
Li et al. (51)	2011–2013	86 (43/43)	38.8 ± 1.3 (w)/38.5 ± 1.2 (w)	CT + Gangliosides (20 mg, qd) + HBOT (–, qd, 0.04–0.08 MPa) (20 mg, qd)	CT + Gangliosides	30 days
Ji (48)	2013–2015	56 (28/28)	38.8 ± 1.4 (w)/38.9 ± 1.3 (w)	CT + Gangliosides (20 mg, qd) + HBOT (50 min, qd, 0.03 MPa)	CT + Gangliosides (20mg, qd)	10 days
Yang (64)	2011–2014	62 (32/30)	–/–	CT + Gangliosides (20 mg, qd) + HBOT (30 min, qd, 0.03–0.04 MPa)	CT + Gangliosides (20 mg, qd)	14 days
Wei and Guo (61)	2012–2014	85 (45/40)	37.48 ± 4.59 (w)/37.95 ± 4.86 (w)	CT + Gangliosides (20 mg, qd) + HBOT (60 min, qd, 0.03–0.04 MPa)	CT	20 days
Du et al. (31)	2014–2017	106 (53/53)	37.01 ± 3.31 (w)/37.55 ± 3.12 (w)	CT + Gangliosides (2 ml, qd) + HBOT (60 min, qd, 0.04–0.08 MPa)	CT	14 days
Meng (55)	2011–2013	50 (25/25)	–/–	CT + Gangliosides (20 mg, qd) + HBOT (50 min, qd, 0.03 MPa)	CT	28 days
He (34)	2013–2015	124 (62/62)	39 ± 3.8 (w)/39 ± 3.8 (w)	CT + Gangliosides (20 mg, qd) + HBOT (50 min, qd, 0.03–0.04 MPa)	CT	20–30 days
Dai (30)	2013–2015	120 (62/58)	37–42 (w)/37–42 (w)	CT + Gangliosides (20 mg, qd) + HBOT (50 min, qd, 0.04–0.08 MPa)	CT	30–50 days
Zhang (71)	2016–2017	88 (44/44)	39.5 ± 0.3 (w)/39.2 ± 0.5 (w)	CT + Gangliosides (20 mg, qd) + HBOT (60 min, qd, –)	CT	20 days
Ni (56)	2015–2017	98 (49/49)	37.61 + 3.4 (w)/37.52 + 3.14 (w)	CT + Gangliosides (2 ml, qd) + HBOT (50–70 min, qd, 0.04–0.08 MPa)	CT	20 days
Yang (65)	–	68 (34/34)	40.2 ± 2.1 (w)/41.1 ± 2.3 (w)	CT + Gangliosides (20 mg, qd) + HBOT (–, qd, 0.040.08 MPa)	CT	10 days
Wang (39)	2013–2014	129 (65/64)	40.4 ± 1.6 (w)/39.6 ± 1.7 (w)	CT + Gangliosides (20 mg, qd) + HBOT (–, qd, 0.04–0.08 MPa)	CT	10 days
Cao (42)	2013–2016	70 (35/35)	1.21 + 0.26 (d)/1.52 ± 0.25 (d)	CT + Gangliosides (20 mg, qd) + HBOT (60 min, qd, 0.03–0.04 MPa)	CT	10–30 days
Zhang (71)	2017–2018	60 (30/30)	40.2 ± 2.0 (w)/40.5 ± 2.2 (w)	CT + Gangliosides (20 mg, qd) + HBOT (–, qd, 0.03–0.06 MPa)	CT	10 days
Zhang et al. (73)	2013–2015	96 (60/36)	–/–	CT + Gangliosides (20 mg, qd) + HBOT (–, qd, 0.03–0.06 MPa)	CT	5 days
Gao (45)	2012–2014	100 (50/50)	4.2 ± 0.7 (d)/ 4.2 ± 0.7 (d)	CT + Gangliosides (20 mg, qd) + HBOT (20 min, qd, 0.03–0.05 MPa)	CT	-
Shao (57)	2011–2013	92 (46/46)	–/–	CT + Gangliosides + HBOT (40 min, qd, 0.03–0.06 MPa)	CT	10 days
Zhang et al. (73)	2013–2015	76 (38/38)	39.2 ± 5.1 (w)/39.7 ± 4.8 (w)	CT + Gangliosides (20 mg, qd) + HBOT (70 min, qd, 0.03–0.04 MPa)	CT	14 days
Zhang et al. (73)	2013–2015	148 (74/74)	39.48 ± 2.51 (w)/39.33 ± 2.64 (w)	CT + Gangliosides (20 mg, qd) + HBOT (45 min, qd, 0.03–0.04 MPa)	CT	20 days
Chao (43)	2017–2018	60 (30/30)	39.2 ± 3.4 (w)/39.1 ± 3.7 (w)	CT + Gangliosides (20 mg, qd) + HBOT (60 min, qd, 0.03–0.04 MPa)	CT	14 days
Du (45)	2012–2013	104 (53/51)	40.1 ± 4.4 (w)/39.4 ± 4.8 (w)	CT + Gangliosides (20 mg, qd) + HBOT (60 min, qd, 0.03–0.04 MPa)	CT	14 days
Liu (54)	2013–2016	60 (30/30)	39.31 ± 1.53 (w)/39.3 ± 1.55 (w)	CT + Gangliosides (20 mg, qd) + HBOT (70 min, qd, 0.03–0.04 MPa)	CT	14 days
Chen (16)	2015–2017	186 (93/93)	3.5 ± 0.6 (d)/4.2 ± 0.8 (d)	CT + Gangliosides (20 mg, qd) + HBOT (60 min, qd, 0.03 MPa)	CT	28 days
Gao (90)	2018–2019	76 (38/38)	37.4 ± 2.5 (w)/37.7 ± 2.9 (w)	CT + Gangliosides (20 mg, qd) + HBOT (60 min, qd, 0.04–0.08 MPa)	CT	14 days
Cai (41)	2013–2016	90 (45/45)	3.52 ± 1.87 (d)/4.06 ± 0.91 (d)	CT + Gangliosides (20 mg, qd) + HBOT (45 min, qd, 0.03–0.04 MPa)	CT	30 days
Li (37)	2015–2018	120 (60/60)	36.78 ± 4.52 (w)/37.03 ± 3.87 (w)	CT + Gangliosides (20 mg, qd) + HBOT (90 min, qd, 0.06 MPa)	CT	28 days

*E, experiment group; C, control group; HBOT, hyperbaric oxygen therapy; CT, conventional treatment.

**FIGURE 2:**
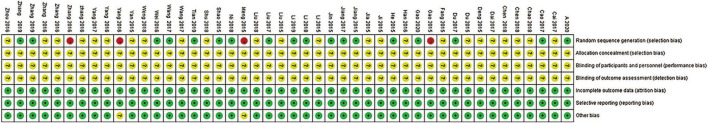
Specific results of deviation assessment.

**FIGURE 3:**
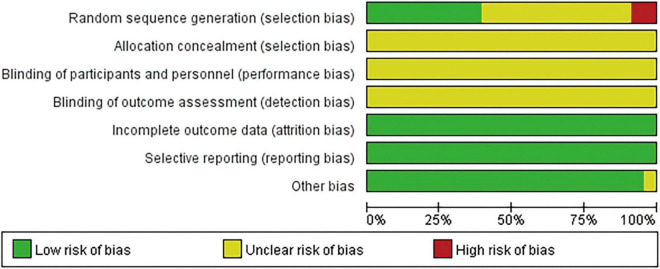
Schematic diagram of methodological quality assessment of the literature in this study.

We found 46 studies eligible for the meta-analysis. Among these, 18 trials (28–45) were performed using random number tables, lottery grouping, and envelope randomization to generate random sequences. Therefore, we concluded that these studies were low-risk. Four trials (48–51) were judged to be high risk due to using non-standard randomization methods based on the treatment regimen. The remaining 24 studies did not provide detailed information about the randomization methods used for the study. Most studies did not provide specific allocation concealment, operator/patient blindness, result evaluator blindness, and random sequence generation. All studies provided complete data on the outcomes, and the risks were found low. The results of the bias assessments are summarized in Figures 2, 3.

### Primary Outcomes

#### Total Efficiency

**FIGURE 4:**
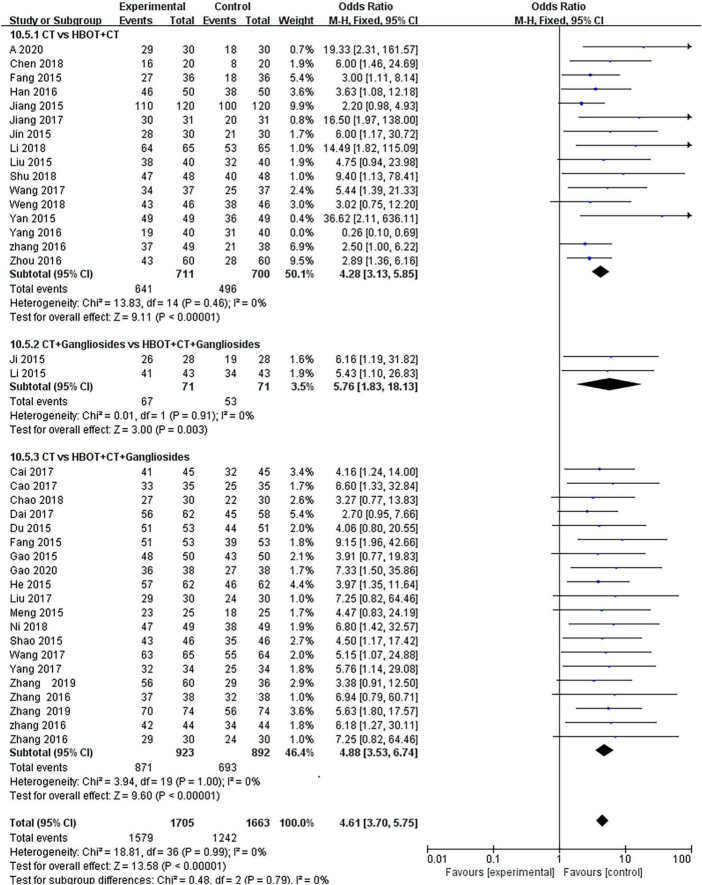
Subgroup analysis of TEF: (1) CT vs. HBOT + CT; (2) CT + Gangliosides vs. HBOT + CT + Gangliosides; (3) CT vs. HBOT + CT + Gangliosides.

Thirty-eight studies (28, 30–34, 36–43, 47–51) compared the effect of conventional treatment and HBOT on the TEF of neonatal HIE patients. There were 3,368 patients with neonatal HIE, specifically 1,579 patients in the HBOT group and 1,242 in the conventional treatment group. The results of the heterogeneity test indicated that there was heterogeneity between the studies (*P* = 0.09; *I*^2^ = 24%), which disappeared (*P* = 0.99; *I*^2^ = 0%) after the study by Ni (56) was removed. The effect sizes were combined with the fixed-effects model, and the results showed that the differences between the two groups were statistically significant [OR = 4.61, 95% CI (3.70.5.75), *P* < 0.00001]. It shows that the effect of HBOT neonatal HIE is significantly better than conventional treatment. The subgroup analysis showed no statistical difference (*P* = 0.79, *I*^2^ = 0%) (Figure 4).

#### Risk of Sequelae

**FIGURE 5:**
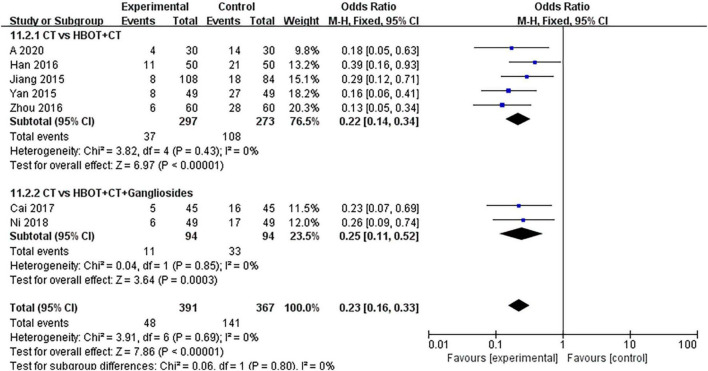
Subgroup analysis of risk of sequelae: (1) CT vs. HBOT + CT; (2) CT vs. HBOT + CT + Gangliosides.

Seven studies (32, 34, 39, 50, 54, 55, 69) compared the effects of conventional treatment and HBOT with the risk of sequelae in neonates with HIE. Long-term follow-up was conducted in two trials (33, 35), for 1 year and 30 months, respectively. Follow-up durations were not specified for the other included trials. Seven hundred fifty-eight neonates with HIE were enrolled, specifically 391 patients in the HBOT group and 367 in the conventional treatment group. The heterogeneity test results showed no heterogeneity between the studies (*P* = 0.69; *I*^2^ = 0%); therefore, the effect sizes were combined with the fixed-effects model. The results showed that there was a statistically significant difference between the two groups [OR = 0.23, 95% CI (0.16, 0.33), *P* < 0.00001]. This indicated that HBOT was superior to the conventional treatment in reducing the risk of sequelae in neonatal HIE patients. No statistical differences were observed in the subgroup analysis (*P* = 0.80, *I*^2^ = 0%) (Figure 5).

#### Neonatal Behavioral, Neurological Assessment Scores

**FIGURE 6:**
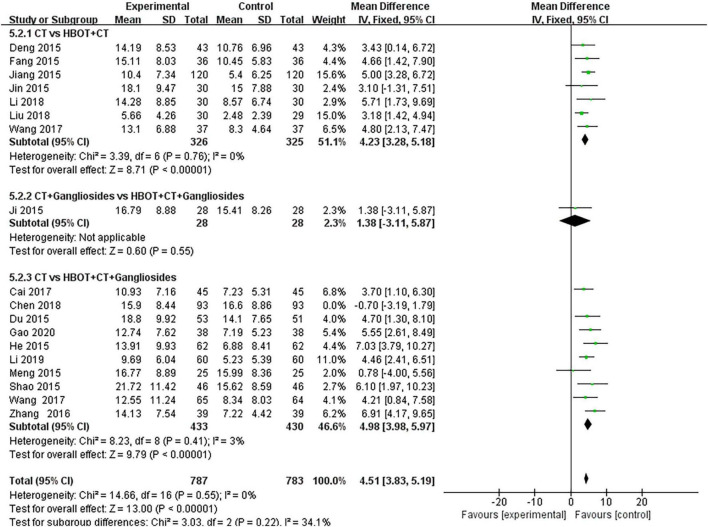
Subgroup analysis of NBNA score: (1) CT vs. HBOT + CT; (2) CT + Gangliosides vs. HBOT + CT + Gangliosides; (3) CT vs. HBOT + CT + Gangliosides.

Eighteen studies (19–24, 28–33, 37, 42, 44, 45, 47, 57, 61, 64, 66, 68–72, 74) compared the effects of conventional treatment and HBOT on the NBNA scores of patients with neonatal HIE. There were 1,570 cases of neonatal HIE, with 880 cases in the HBOT group and 876 cases in the conventional treatment group. The results of the heterogeneity test showed that there was heterogeneity between the studies (*P* = 0.02; *I* = 44%), but no heterogeneity was found after the removal of the study by Zhang (72) (*P* = 0.55; *I*^2^ = 0%). The differences between the two groups were statistically significant [MD = 4.51, 95% CI (3.83, 5.19), *P* < 0.00001]. This indicated that HBOT was superior to conventional therapy in improving the NBNA scores of neonates with HIE. No statistically significant differences were observed in the subgroup analysis (*P* = 0.22, *I*^2^ = 34.1%) (Figure 6).

### Publication Bias

#### Heterogeneity Test

For the 46 reference articles (28–73) evaluated in this study, *I*^2^ = 22% < 25% after the heterogeneity test and *P* = 0.1 for the Q test, suggesting a minor possibility of heterogeneity among the reference articles. A fixed-effects model was used for meta-analysis. To ensure the accuracy and stability of the study, sensitivity analysis was performed.

#### Sensitivity Analysis

**FIGURE 7:**
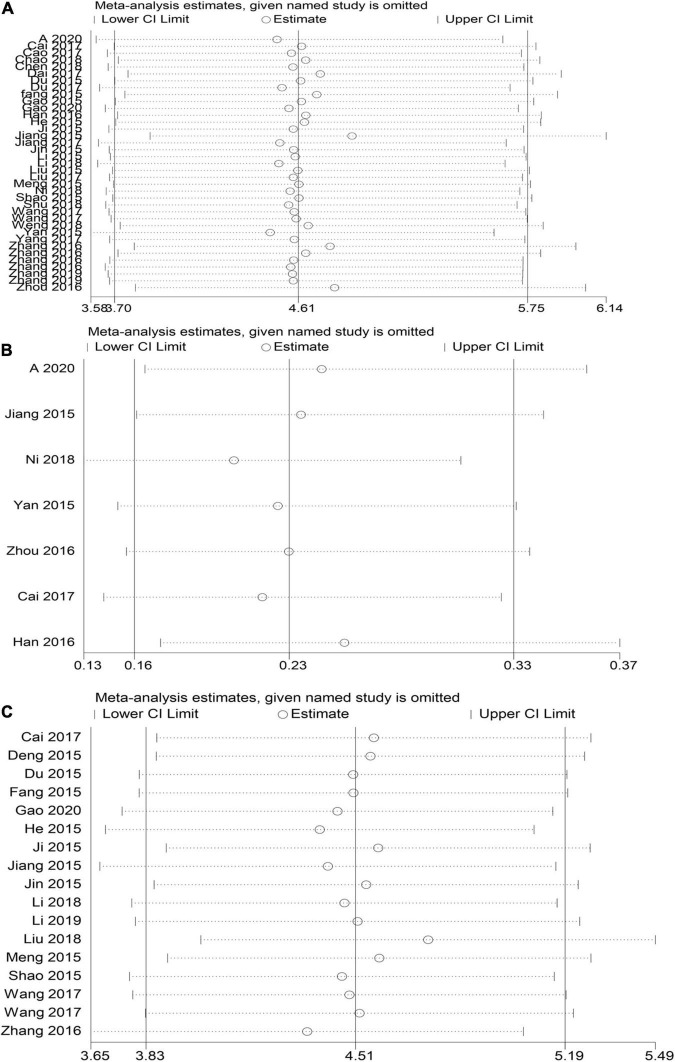
HBOT and conventional treatment of neonates with HIE sensitivity analysis curve. **(A)** TEF, **(B)** risk of sequelae, **(C)** NBNA score).

STATA15 was used for sensitivity analysis of the key outcome indicators, specifically TEF, the risk of sequelae, and the NBNA score. The results showed that the elimination of each outcome and individual studies did not significantly alter the meta-analysis results, indicating that the study had good stability; hence, the study results were considered reliable (Figure 7).

#### Bias Test

**FIGURE 8:**
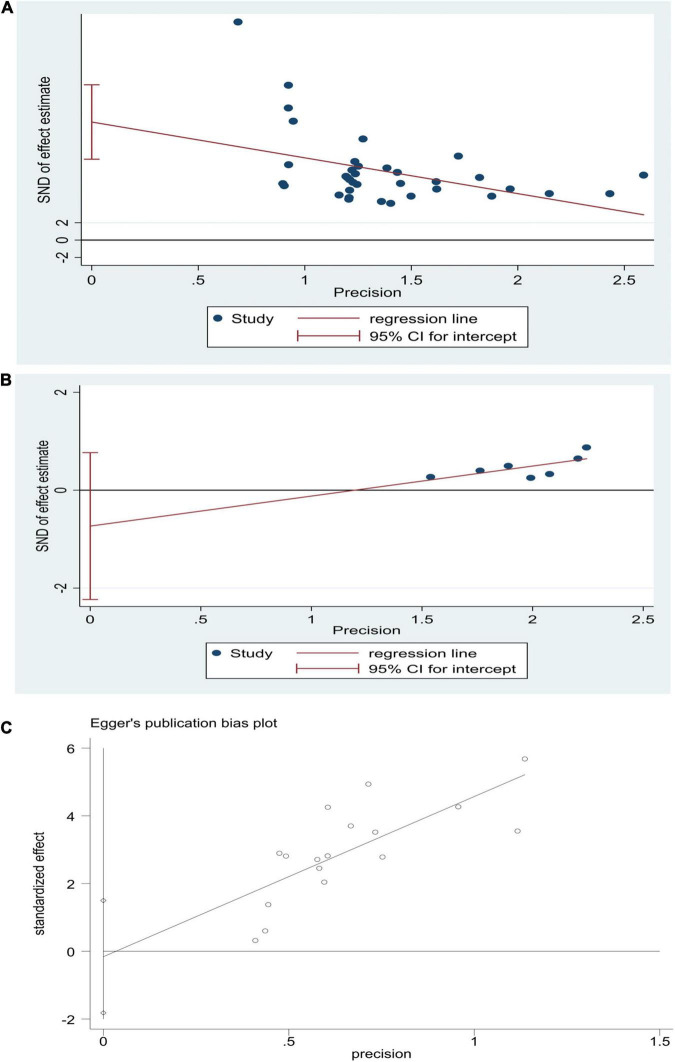
Egger’s publication bias graphs for HBOT and conventional treatment of neonatal HIE. **(A)** TEF, **(B)** risk of sequelae, **(C)** NBNA score.

**FIGURE 9:**
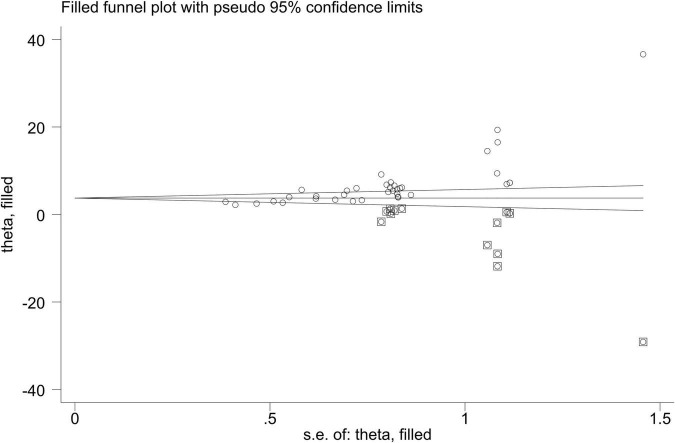
The filling funnel plot of the TEF of HBOT and conventional treatment for neonatal HIE.

STATA15 was used to test the publication bias for the major outcome indicators. If the preliminary results were likely biased, i.e., *P* < 0.05, significant publication bias was evaluated with the shear complement method. According to Egger’s inspection results, there was no publication bias in the risk of sequelae [*P* > |*t*| = 0.264, 95% CI (−2.23, 0.77)] and the NBNA score [*P* > |*t*| = 0.422, 95% CI (−2.14, 4.86)] in the two groups before and after the treatment. There was significant publication bias [*P* > |*t*| = 0.0001, 95% CI (9.85, 18.32)] (Figure 8) in the TEF. The TRIM and padding method was used to evaluate the reliability of the results affected by the significant publication bias. The OR and 95% CI after dressing and filling [OR = 3.47, 95% CI (3.27, 3.67), *P* < 0.00001] were consistent with the previous results [OR = 4.61, 95% CI (3.70, 5.75), *P* < 0.00001], indicating that the results were reliable (Figure 9). The possibility of publication bias is mainly based on small studies’ (over-) presence with (very) high effect sizes. Small studies have large standard errors, and only those with very high effect sizes, thus overcoming their standard error, are deemed to get published.

## Discussion

Neonatal HIE is a clinical syndrome caused by brain injury during the perinatal period, in the weeks leading up to birth, or during labor and delivery. This is caused by partial or complete hypoxia in infants during perinatal asphyxia, resulting in the reduction or suspension of cerebral blood flow, which is the main cause of hypoxia (75, 76). Early diagnosis and timely intervention are of great significance to improve the prognosis of neonatal HIE and reduce the mortality of children. The diagnostic methods of neonatal HIE mainly include ultrasound, computed tomography, MRI, etc. Among them, MRI has high spatial resolution and soft-tissue resolution, judging intracranial lesions and reducing radiation damage. It is the current neonatal ischemic deficiency. The best way to check oxygen encephalopathy (77). In some instances, the appropriate diagnosis is essential within 24 h to reduce the mortality rate. According to research, to date, there is no specific therapy for neonatal HIE treatment. The existing treatment methods mainly include support for symptomatic treatment, mild hypothermia treatment, EPO, xenon, stem cell therapy, etc. (14, 17, 78, 79). However, current treatments focused on reducing the occurrence of sequelae, reducing mortality and neuroprotection are unsatisfactory. Therefore, finding clinically effective complementary therapies with lower adverse events can improve the efficacy of conventional treatments in the treatment of neonatal HIE.

Studies have shown that hypoxia is the main pathological factor leading to brain nerve cell function damage. The use of HBOT has gradually increased as it is a non-invasive method that involves inhaling pure oxygen in a pressurized chamber with high levels of atmospheric pressure. In HBOT of patients with brain injury, tracheal intubation is an effective way of inhaling oxygen, which continuously injects hyperbaric oxygen into the patient’s body. It can increase blood oxygen content, increase blood oxygen partial pressure, increase oxygen diffusion distance, and by doing so, it corrects the hypoxic state of brain tissue (80). Furthermore, HBOT activates the lungs by providing oxygen to other systemic organs, minimizing secondary brain injury events like rampant inflammation, apoptosis initiation, and oxidative stress (81). The partial pressure of oxygen in the alveoli induced by pressurized oxygen increases the oxygen level of brain tissue, reduces the energy failure of brain tissue, inhibits cellular apoptosis, and reduces brain damage, which can minimize brain nerve damage from HIE (14, 81). An earlier animal study showed that HBOT can enhance the proliferation of neural stem cells in the subventricular region of HIE neonatal rats and has the therapeutic potential to promote nervous system recovery after brain injury (82). At the cellular level, hyperbaric oxygen preconditioning can increase the expression of Nrf2 and the activity of downstream proteins to reduce hypoxic-ischemic brain injury, which significantly reduces the infarct area, neuronal injury, and cell apoptosis (21). Liu et al. (83) and Zhang et al. (84) published systematic evaluations of neonatal HIE treated with HBOT in China. Both studies showed that HBOT reduced the mortality rate and neurological sequelae in neonatal HIE. However, due to the lack of inadequate information in the literature, the results are likely to be biased; hence, the evidence may be unreliable, and further investigation is required. The studies also did not provide details of the treatment strategy or protocol information, and therefore, subgroup analysis could not be performed according to the HBOT protocol. Hence, this meta-analysis aimed to evaluate the efficacy and prognosis of HBOT for HIE quantitatively. The results obtained suggest that HBOT for HIE is more effective than the routine, conventional treatment and can effectively improve the neurological function of HIE children and efficiently reduce the risk of neurological sequelae. Our results were in agreement with the meta-analysis by Zhang and Liu. We also described detailed strategies and schemes for HBOT in neonatal HIE for the first time by providing more reliable clinical evidence-based HBOT for neonatal HIE in clinical practice.

This study evaluated evidence from 46 RCTs, and a total of 4,199 neonatal HIE patients were randomized to receive HBOT or conventional treatment from 2015 to 2020. The results of the study are as follows: (1) TEF of the HBOT group is significantly better than that of the CT group; (2) the HBOT group is significantly better than the CT group in reducing the incidence of sequelae in children; (3) the NABA score of the HBOT group was significantly higher than that of the CT group. So the benefits of HBOT are obvious. Although this study proved that HIE in neonates with HBOT is effective, the included studies have not reported on mortality, so the safety of hyperbaric oxygen therapy in neonates with HIE has aroused everyone’s attention. A meta-analysis of 51 animal studies confirmed that the HBOT group exhibited a 32% reduction in the cerebral infarction area compared to the control group. Significant improvements were observed in neurological function [95% CI (28–37%), *P* < 0.00001] and the mortality rate decreased by 8.3% (85). In addition, as early as 2016, a meta-analysis by Wang F showed that HBOT plays an important role in traumatic brain injury and can significantly improve the Glasgow coma scale (GCS) and Glasgow outcome score (GOS) for patients as well as reduce disability and mortality (86). However, prolonged hyperbaric oxygen exposure may lead to oxygen poisoning, causing damage to multiple organs of the body. When the partial pressure of oxygen is > 3 ATA, oxygen poisoning may occur. The severity of oxygen poisoning is closely related to the length of exposure (87). However, as long as the pressure and time of hyperbaric oxygen treatment are strictly controlled, the probability of oxygen poisoning is extremely small. Studies have shown that HBOT pressures of 1.4, 1.5, and 1.6 ATA are safe and effective for neonatal HIE (18, 88). Clinically, the following methods can be used to prevent the occurrence of oxygen poisoning: (1) Strictly follow the treatment strategy for treatment and do not increase the treatment pressure arbitrarily. (2) Strictly control the treatment time, adopt the intermittent oxygen inhalation method of inhaling oxygen, inhaling air at rest, and inhaling oxygen again (87).

To determine the best treatment scheme for HBOT neonates with HIE, we conducted a subgroup analysis of the total effective rate according to the daily oxygen uptake, treatment duration, and pressure with HBOT. The results showed that the therapeutic effect for the group with daily oxygen intake for 30–40 min was higher than that for the group with daily oxygen intake for 50–60 min and 70–90 min. This suggests that daily oxygen intake for 30–40 min provides the maximum therapeutic effect for patients with neonatal HIE. Subgroup analysis of the treatment duration showed that the therapeutic effect with a treatment duration of over 30 days was higher than that with a treatment duration of 1–10 days and 10–30 days, suggesting that HBOT over 30 days provides the maximum therapeutic effect for patients with neonatal HIE. The pressure subgroup analysis for HBOT showed that pressure between 0.04 and 0.08 mpa had the best therapeutic effect on neonatal HIE patients. Therefore, based on the results of subgroup analysis findings of HBOT for neonatal HIE patients, treatment could be as follows: daily oxygen uptake for 30–40 min at a pressure of 0.04–0.08 mpa, with the treatment lasting for more than 30 days (Table 2). However, due to the heterogeneity of studies protocol, more research is needed before this potential therapy for HIE can be widely used. Furthermore, because the patients selected for the study are mainly from China, the conclusions of this meta-analysis may not apply to other ethnic groups.

**TABLE 2:** Subgroup analysis of oxygen uptake, treatment duration, and HBOT pressure.

Subgroups	Trials	Effects model	Pooled effect	95% CI	*P*-value
**TEF**					
Oxygen intake (30–40 min, qd)	2	Fixed	7.15	2.35, 21.75	0.0005
Oxygen intake (50–60 min, qd)	22	Fixed	4.56	3.39, 6.13	<0.00001
Oxygen intake (70–90 min, qd)	4	Fixed	4.69	3.16, 9.44	<0.0001
Total	28	Fixed	4.71	3.61, 6.13	<0.00001
**Test for subgroup differences: Chi^2^ = 0.59. df = 2 (*P* = 0.75). *I*^2^ = 0%**					
Treatment duration (1–10 days)	8	Fixed	4.80	1.93, 7.87	<0.00001
Treatment duration (10–30 days)	15	Fixed	4.39	3.10, 6.22	<0.00001
Treatment duration (more than 30 days)	11	Fixed	5.23	3.51, 7.78	<0.00001
Total	34	Fixed	4.75	3.77, 5.99	<0.00001
**Test for subgroup differences: Chi^2^ = 0.42. df = 2 (*P* = 0.81). *I*^2^ = 0%**					
HBOT pressure (0.03–0.04 mpa)	12	Fixed	4.45	2.97, 6.67	<0.00001
HBOT pressure (0.04–0.08 mpa)	12	Fixed	5.06	3.39, 7.57	<0.00001
Total	24	Fixed	4.75	3.57, 6.32	<0.00001
**Test for subgroup differences: Chi^2^ = 0.2. df = 1 (*P* = 0.66). *I*^2^ = 0%**					

This study had the following limitations: (i) We searched only English and Chinese databases. (ii) Some of the included studies did not specify the blinding method used, which may have impacted the objectivity of the neonatal HIE results, leading to measurement bias. (iii) The quality of some included studies was low. Most of the articles did not describe the randomization method used or the concealment of randomly assigned schemes; hence, there may have been a selection bias. (iv) Most of the selected studies in this study had a small sample size and a low design quality, which may have affected the efficacy of HBOT. (v) We have not included any other standard of care such as moderate hypothermia as we believe that these are not in a competitive relationship with HBOT, and they play different roles through different pathways at different stages of the disease and together promote the recovery of the disease (89). However, although the deficiencies listed above may affect the quality of the evidence, the included studies were highly comparable, and the articles were selected with relatively strict inclusion criteria. Therefore, this study highlights the shortcomings of existing studies and can indicate a direction for future studies. The present study has value for clinical research and application and can provide reliable evidence for the effectiveness and prognosis of HBOT for neonatal HIE.

## Conclusion

This meta-analysis showed that the addition of HBOT to the standard and conventional treatment of neonatal HIE significantly improved the children’s NBNA score and clinical efficacy and reduced the risk of sequelae. In light of the effectiveness of HBOT, it holds promise as a potential complementary treatment for neonatal HIE. However, due to the heterogeneity of studies protocol, more research is needed to understand its potential as a therapy for HIE. It is worth noting that because the patients selected for the study are mainly from China, the conclusions of this meta-analysis may not apply to other ethnic groups.

## Data Availability Statement

The raw data supporting the conclusions of this article will be made available by the authors, without undue reservation.

## Author Contributions

JX, Q-LD, R-HF, and X-BG had full access to all the data in the study and took responsibility for the integrity of the data and the accuracy of the data analysis. X-BG and JX contributed to design, statistical analysis, supervision, and manuscript drafting. H-MD and Q-LD contributed to the conception and design, data analysis, funding, supervision, and critical revision of the manuscript. Y-NG, R-HF, and W-HL participated in the acquisition and analysis of data, statistical analysis, and manuscript revision. X-YJ and Y-HL contributed to the analysis and interpretation of the data and revision of the manuscript. All authors read and approved the final manuscript.

## Conflict of Interest

The authors declare that the research was conducted in the absence of any commercial or financial relationships that could be construed as a potential conflict of interest.

## Publisher’s Note

All claims expressed in this article are solely those of the authors and do not necessarily represent those of their affiliated organizations, or those of the publisher, the editors and the reviewers. Any product that may be evaluated in this article, or claim that may be made by its manufacturer, is not guaranteed or endorsed by the publisher.
